# *GST* polymorphism as a predictive biomarker for modulating the susceptibility to chronic obstructive pulmonary disease: A North Indian study

**DOI:** 10.1113/EP091339

**Published:** 2023-11-10

**Authors:** Harsh Yadav, Depanshi Pandit, Sidhartha Singh, Parul Sharma, Kranti Garg, Nidhi Girdhar, Karan Sharma, Vishal Chopra, Siddharth Chopra, Siddharth Sharma

**Affiliations:** ^1^ Department of Pulmonary Medicine Government Medical College TB & Chest Diseases Hospital Patiala Punjab India; ^2^ Department of Biotechnology Thapar Institute of Engineering and Technology Patiala Punjab India; ^3^ Department of Internal Medicine St. Joseph Mercy Oakland Hospital Pontiac Michigan USA

**Keywords:** COPD, GSTM1, GSTT1, multiplex PCR, polymorphism

## Abstract

Chronic obstructive pulmonary disease (COPD) is commonly characterized by shortness of breath, coughing or expectoration. Smoking is the leading cause of COPD development, but only a small percentage of smokers develop symptoms, implying a genetic component. Glutathione *S*‐transferase enzymes are responsible for detoxifying cigarette smoke components. The role of glutathione *S*‐transferase T1 (GSTT1) and glutathione S‐transferase M1 (GSTM1) gene polymorphism was assessed with COPD susceptibility and associated clinical parameters in the North Indian population. This was a cross‐sectional study involving 200 COPD patients and 200 healthy individuals, with peripheral blood sampling and adequate questionnaires. Multiplex PCR was used for genotyping *GSTT1* and *GSTM1* gene polymorphism. Logistic regression was used to calculate the odds ratio and 95% confidence intervals to assess the COPD risk and *GST* polymorphisms. The *GSTT1* gene deletion rate was higher in COPD cases (34.5%) than in healthy individuals (20.5%). A statistical relationship between the *GSTT1*(−) null genotype and COPD risk was observed (odds ratio = 2.04, 95% CI = 1.30–3.20, *P* = 0.0019). After adjusting for covariates like age, sex and smoking status, a significant association was found for *GSTT1*(−) null genotype and COPD risk (adjusted odds ratio = 2.90, 95% CI = 1.43–5.87, *P* = 0.003). The *GSTT1*(−) genotype was also significantly correlated with clinical parameters for COPD risk. Another primary observation was that females with the *GSTT1*(−) null genotype were more vulnerable to COPD than males with the same gene deletion. The *GSTT1*(−) null genotype strongly correlates with COPD development, while no association was observed in the *GSTM1*(−) null genotype in the North Indian population.

## Abstract (highlights)

- **What is the central question of this study?**
  Genetic variations in the GSTT1 and GSTM1 enzymes that detoxify cigarette smoke products might be correlated to chronic obstructive pulmonary disease (COPD) development: do *GSTT1* and *GSTM1* polymorphisms modulate the risk for COPD in North Indians and what is the relationship with associated clinical parameters?
- **What is the main finding and its importance?**
  The *GSTT1*(−) null genotype was strongly correlated with COPD development, and *GSTT1* deletion was also linked with higher Global Strategy for Obstructive Lung Disease (GOLD) grading. At the same time, no association was observed in the *GSTM1*(−) null genotype in the North Indian COPD patients.

## 1 INTRODUCTION

Chronic obstructive pulmonary disease (COPD) is the second leading cause of death and disability‐adjusted life years (DALYs) in India, according to the Global Burden of Disease report (Salvi & Ghorpade, 2021). It is a significant public health problem and is currently a leading cause of morbidity and mortality worldwide, leading to a considerable rise in the social and economic burden (Lozano et al., 2012; Vos et al., 2012). Tobacco smoke exposure or tobacco smoking is one of the most critical factors leading to COPD, in addition to genetic and environmental factors.

Studies in the past suggest that genetic variations in the enzymes that detoxify cigarette smoke products might be correlated to COPD development. These oxidation‐inhibiting enzymes include microsomal epoxide hydrolase, cytochrome p450 1A1 and glutathione *S*‐transferase (GST) (Yim et al., 2000). Genetic variation reduces the production or activity of such oxidation‐inhibiting enzymes, and the dynamic balance of oxidation/antioxidation is lost, resulting in oxidative damage (Zhang et al., 2015). *GST* is one of the most studied genes in various human populations, and GST is involved in the metabolism of endogenous and environmental xenobiotics and plays a role in the susceptibility to COPD (Lakhdar et al., 2011). GST is a detoxifying phase II biotransformation enzyme. It is classified as a cytoplasmic, membrane, mitochondrial or leukotriene C4 synthase. There are eight mammalian classes of these enzymes represented by the Greek letters α, μ, π, σ, θ, κ, ζ and ω (Nebert & Vasiliou, 2004). The conjugating reaction of reduced glutathione and GSTs eliminates the harmful electrophilic compounds formed by oxidative stress (Rezaei et al., 2013). The enzyme is required for organisms to respond to physiochemical stimulation in the environment and protect cells, proteins and nucleic acids from damage caused by free radicals. According to recent research, GST enzymes bind to various toxic molecules from cigarettes, that is, oxidizing agents or free radicals, which act as substrates for biotransformation metabolism, safeguarding cells from carcinogenic and cytotoxic factors (Sharma et al., 2015; Tang et al., 2013).

The *GSTM1* and *GSTT1* genes are expressed in the respiratory tract. Studies have revealed that *GSTM1* and *GSTT1* gene deletions may be linked to the onset and progression of COPD (Chirilă et al., 2014; Kant Shukla et al., 2013; Lakhdar et al., 2010, 2011). GSTT1 is responsible for detoxifying conjugated lipid peroxide and halogenated compounds, whereas GSTM1 is responsible for detoxifying benzopyrene diol epoxide (Lakhdar et al., 2011). *GSTM1* and *GSTT1* are located on chromosomes 1p13.3 and 22q11.23, respectively. Individuals with *GSTM1* null or *GSTT1* null genotypes have reduced or no GST enzyme activity, making them more vulnerable to various factors that cause COPD. Diabetes mellitus, rheumatoid arthritis, systemic sclerosis, bronchial asthma, Parkinson's disease and various cancers have been linked to *GSTM1* and *GSTT1* gene deletions (Birbian et al., 2012; Chirilă et al., 2014; Kim, 2017; Krüger et al., 2015; Mastana et al., 2013; Pinhel et al., 2013; Safarinejad et al., 2013; Sharma et al., 2015; Tew et al., 2001).

Gene deletion is the primary cause of *GSTM1* and *GSTT1* null alleles (Lakhdar et al., 2010). Individuals with *GSTM1* and *GSTT1* homozygous null alleles lack the corresponding enzyme function, increasing susceptibility to COPD. COPD susceptibility has been seen to be polygenic. Several studies have found polymorphisms of these genes in people of different ethnicities with COPD. However, the association of *GSTM1* and *GSTT1* polymorphisms with COPD progression was inconclusive across various nationalities.

The present study was undertaken to investigate the role of *GSTT1* and *GSTM1* polymorphisms as genetic markers for COPD risk in the North Indian population and evaluate the relationship.

## 2 METHODS

### 2.1 Ethical approval

The current study included COPD patients who visited the Department of Pulmonary Medicine, TB & Chest Diseases Hospital, Government Medical College, Patiala, Punjab, India. The research study was given ethical approval by the Baba Farid University of Health Sciences Institute's ethics board (Approval No. BFUHS/2K21p‐TH/5420, dated 28/4/2021). The patients gave informed consent to participate in the study, and a questionnaire for every patient was filled out by trained personnel. The study conformed to the principles of the *Declaration of Helsinki*, except for registration in a database.

### 2.2 Study design

A cross‐sectional study was conducted on 200 COPD patients and 200 healthy controls presenting to the outpatient department of Pulmonary Medicine, Government Medical College, Patiala, Punjab, India. After the clinical history and physical examination, they were subjected to spirometry and were classified according to the clinical criteria of COPD set down in the Global Strategy for Obstructive Lung Disease (GOLD) 2019 guidelines (Gruffydd‐Jones, 2012). Blood samples of all 400 individuals were collected in EDTA‐coated tubes and stored at −20°C for extraction of DNA.

### 2.3 DNA extraction and genotyping

**FIGURE 1:**
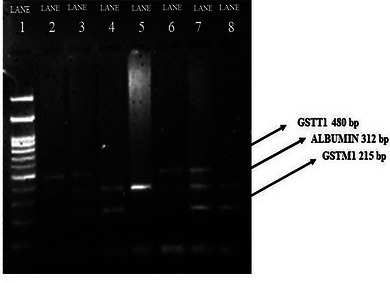
Two percent agarose gel of PCR products for the distribution of GST polymorphism. Lane 1: marker (M) (100 bp); lane 2 and 6: *GSTM1*(−), *Albumin*(+), *GSTT1*(+); lane 3 and 7 *GSTM1*(+), *Albumin*(+), *GSTT1*(+); lane 4 and 8: *GSTM1*(+), *Albumin*(+), *GSTT1*(−); lane 5: *GSTM1*(−), *Albumin*(+), *GSTT1*(−).

Genomic DNA was isolated using standard protein K digestion, phenol–chloroform extraction and ethanol precipitation from whole blood samples. The presence of genomic DNA was checked on 0.8% agarose gel. Multiplex PCR amplifies multiple targets in a single set of reaction conditions using multiple primers. The PCR mixture of 20 μL comprised 1× PCR buffer, 100μg/ml bovine serum albumin, 0.5 μM forward primers and 0.5 μM reverse primers of both *GSTM1* and *GSTT1*, 0.3 μM of forward and reverse primer of *Albumin*, 0.2 μM dNTPs, 3 U/μL of Taq polymerase and 300 ng of DNA. An optimal combination of annealing temperature and buffer concentration was maintained, as is necessary for multiplex PCR to obtain amplified products. The sequence of primers used was as follows: for *GSTM1*, forward: 5′‐GAACTCCCTGAAAAGCTAAAGC‐3′, and reverse: 5′‐GTTGGGCTCAAATATACGGTGG‐3′; for*GSTT1*, forward: 5′ TTCCTTACTGGTCCTCACATCTC‐3′, and reverse: 5′‐TCACCGGATCATGGCCAGCA‐3′; and for the albumin gene, forward: 5′‐GCCCTCTGCTAACAAGTCCTAC‐3′, and reverse: 5′‐CCCTAAAAAGAAAATCGCCAATC‐3′. The PCR was done as follows: initial 95°C for 5 min, 30 cycles of denaturation (94°C, 1 min), annealing (59°C, 1 min) and elongation (72°C, 1 min), and a final elongation step at 72°C for 5 min. The PCR product was then subjected to 2.0% agarose gel electrophoresis. A band of size 480 bp indicated the *GSTT1* allele, and that of 215 bp indicated the *GSTM1* allele. The absence of both 215 bp and 480 bp bands indicated the null genotype. *Albumin* was an internal control indicated by a band of size 312 bp (Figure 1).

### 2.4 Statistics

Differences in the distributions of demographic characteristics between the COPD cases and healthy individuals were evaluated using the Chi‐square (χ^2^) test for the categorical data and Student's *t*‐test for continuous variables. The Hardy–Weinberg equilibrium theory (*p* ^2^ + 2*pq* + *q* ^2^ = 1, where *p* is the frequency of the wild‐type allele and *q* is the frequency of the variant allele) was used both in COPD cases and healthy individuals to calculate the genotype frequencies of *GSTM1* and *GSTT1* gene polymorphism using the χ^2^ test. Pearson's χ^2^ test was used to determine whether there was any significant difference in allele and genotype frequencies between COPD cases and healthy controls. To assess the association between COPD and GST polymorphisms, adjusted odds ratios (ORs) and 95% confidence intervals (CI) were calculated. Logistic regression analysis was applied to adjust possible confounders (age and pack‐years of smoking as continuous variables and sex as a nominal variable). All *P*‐values were two‐sided, and a *P*‐value of <0.05 was considered statistically significant. The statistical analysis was performed with MedCalc version 9.3.6.0 (MedCalc Software, Ostend, Belgium) and SPSS Statistics Version 20.0 (IBM Corp., Armonk, NY, USA).

## 3 RESULTS

### 3.1 Demographic and clinical characteristics of COPD patients and healthy controls

The COPD patients and healthy controls had a mean age of 59.3 ± 9.9 (range 41–80) and 47 ± 15.6 (range 21–92) years, respectively. The COPD group consisted of 183 (91.5%) males and 17 (8.5%) females, while the healthy control group included 115 (57.5%) males and 85 (42.5%) females. The mean body mass index (BMI) of COPD cases was 21.7 ± 5.3 kg/m^2^, and the BMI in healthy controls was 25.3 ± 4.7 kg/m^2^. The mean number of pack‐years of smoking in the study was 49.6. The categorization of the cases as per GOLD severity based on forced expiratory volume in 1 s (FEV1) spirometric readings is given in Table 1. Shortness of breath was the most common symptom with which the patients presented to us (Table 1).

**TABLE 1:** Demographic and clinical characteristics among COPD patients and healthy controls.

Parameter	Cases (*n* = 200)	Controls (*n* = 200)	*P*
Males (*n* (%))	183 (91.5)	115 (57.5)	**0.0001**
Females (*n* (%))	17 (8.5)	85 (42.5)	
Age (mean ± SD, years)	59.3 ± 9.9	47 ± 15.6	**0.0001**
BMI (mean ± SD, kg/m^2^)	21.7 ± 5.3	25.3 ± 4.7	
Pack years (mean)	49.6		**0.034**
GOLD 1 (mild) (*n* (%))	7 (3.5)		**0.0001**
GOLD 2 (moderate) (*n* (%))	71 (35.5)		
GOLD 3 (severe) (*n* (%))	79 (39.5)		
GOLD 4 (very severe) (*n* (%))	43 (10.8)		
Shortness of breath (*n* (%))	196 (98)		**0.0001**
Cough (*n* (%))	39 (19.5)		
Expectoration (*n* (%))	31 (15.5)		
Others (*n* (%))	3 (1.5)		

### 3.2 Distribution and association of *GST* polymorphism with COPD risk

The *GSTT1*(−) null genotype prevalence was 34.5% in COPD patients and 20.5% in healthy individuals. When adjusted with covariates such as age, sex and smoking status, a significant association was observed between *GSTT1*(−) null genotype and COPD risk (adjusted odds ratio (AOR) = 2.90, 95% CI = 1.43–5.87, *P* = 0.003). On the contrary, the *GSTM1*(−) null genotype was present in 36.5% of COPD cases and 36.5% of controls. No association was found between the *GSTM1*(−) genotype and COPD risk (odds ratio (OR) = 1.0, 95% CI = 0.66−1.50, *P* = 1) (Table 2).

**TABLE 2:** Distribution and association of *GST* polymorphism with COPD risk.

Genotype	Controls (*n* (%)) (*n* = 200)	Cases (*n* (%)) (*n* = 200)	OR (95% CI)	*P*	AOR (95% CI)	*P*
GSTM1						
*GSTM1*(+)	127 (63.5)	127 (63.5)	1.00 (reference)	–	1.00 (reference)	–
*GSTM1*(−)	73 (36.5)	73 (36.5)	1.0	1	0.98 (0.52–1.84)	0.96
GSTT1						
*GSTT1*(+)	159 (79.5)	131 (65.5)	1.00 (reference)	–	1.00 (reference)	–
*GSTT1*(−)	41 (20.5)	69 (34.5)	2.04 (1.30–3.20)	**0.0019**	2.90 (1.43–5.87)	**0.003**
*GSTM1*(+)/*GSTT1*(+)	96 (48)	75 (37.5)	1.00 (reference)	–	1.00 (reference)	–
*GSTM1*(−)/*GSTT1*(−)	10 (5)	17 (8.5)	2.17 (0.94–5.02)	0.06	2.66 (0.79–8.94)	0.11
*GSTM1*(+)/*GSTT1*(−)	31 (15.5)	52 (26)	2.14 (1.25–3.67)	**0.005**	2.93 (1.29–6.65)	**0.009**
*GSTM1*(−)/*GSTT1*(+)	63 (31.5)	56 (28)	1.13 (0.71–1.82)	0.59	1.22 (0.57–2.61)	0.60

OR, crude odds ratio; AOR, adjusted odds ratio, evaluated by unconditional logistic regression and adjusted for age, sex and smoking. *P*‐values in bold indicate statistical significance (*P* < 0.05).

### 3.3 Combinatorial association of *GST* polymorphism with susceptibility of COPD

Our study evaluated the relationship between COPD risk and different genotypic combinations. The *GSTM1*(+)/*GSTT1*(+) genotype was found in 75 (37.5%) subjects of the COPD cases and 96 (48%) healthy individuals. The *GSTM1*(+)/*GSTT1*(−) genotype was reported in 52 (26%) COPD cases and 31 (15.5%) healthy individuals (OR = 2.14, 95% CI = 1.25–2.67, *P* = 0.005). After adjusting with different covariates such as age, sex and smoking status, the risk increased by 2.9‐fold (AOR = 2.93, 95% CI = 1.29–6.65, *P* = 0.009). The *GSTM1*(−)/*GSTT1*(−) genotype was present in 17 (8.5%) COPD patients and 10 (5%) healthy individuals (OR = 1.13, 95% CI = 0.71–18.2, *P* = 0.59; AOR = 1.22, 95% CI = 0.57–2.61, *P* = 0.60) (Table 2).

### 3.4 Association of *GST* polymorphism and COPD based on sex

In males, the occurrence of the *GSTT1*(−) null genotype was 31% (62) in COPD cases and 13% (26) in healthy individuals. The COPD risk was significantly associated with *GSTT1*(−) null deletion among males (OR = 1.75, 95% CI = 1.02–2.99, *P* = 0.03) (Table 3). In females, the presence of the *GSTT1*(−) null genotype was 3.5% (7) in COPD cases and 7.5% (15) in healthy individuals. A strong correlation between the *GSTT1*(−) null genotype and COPD risk was found among females (OR = 3.26, 95% CI = 1.07–9.96, *P* = 0.03). A seven‐fold increase in COPD susceptibility in females with the *GSTT1*(−) null genotype was observed after adjusting it with factors like age and smoking status (AOR = 7.11, 95% CI = 1.90–26.64, *P* = 0.003) (Table 3).

**TABLE 3:** Association of GST polymorphism and COPD based on sex.

Genotype	Controls (*n* (%)) (*n* = 200)	Cases (*n* (%)) (*n* = 200)	OR (95% CI)	*P*	AOR (95% CI)	*P*
Male						
*GSTM1*						
*GSTM1*(+)	73 (36.5)	116 (58)	1.00 (reference)	–	1.00 (reference)	–
*GSTM1*(−)	42 (21)	67 (33.5)	1.00 (0.61–1.62)	0.98	0.91 (0.433–1.88)	0.79
*GSTT1*						
*GSTT1*(+)	89 (44.5)	121 (60.5)	1.00 (reference)	–	1.00 (reference)	–
*GSTT1*(−)	26 (13)	62 (31)	1.75 (1.02–2.99)	**0.03**	2.04 (0.91–4.59)	0.08
Female						
*GSTM1*						
*GSTM1*(+)	54 (27)	11 (5.5)	1.00 (reference)	–	1.00 (reference)	–
*GSTM1*(−)	31 (15.5)	6(3)	0.95 (0.32–2.82)	0.92	1.24 (0.36–4.24)	0.72
*GSTT1*						
*GSTT1*(+)	70 (35)	10 (5)	1.00 (reference)	–	1.00 (reference)	–
*GSTT1*(−)	15 (7.5)	7 (3.5)	3.26 (1.07–9.96)	**0.03**	7.11 (1.90–26.64)	**0.003**

OR, crude odds ratio; AOR, adjusted odds ratio, evaluated by unconditional logistic regression and adjusted for age, sex and smoking. *P* < 0.05 was considered to be statistically significant.

### 3.5 Association of *GST* polymorphism with modified Medical Research Council dyspnea score and COPD Assessment Test score for COPD risk

We evaluated the relationship of the modified Medical Research Council (mMRC) dyspnea score with GST polymorphism in both COPD patients and healthy individuals. Two groups were categorized based on the cut‐off point of mMRC < 2 and mMRC ≥ 2 as recommended by the GOLD 2011 guidelines. In the first group, that is, mMRC < 2, the *GSTT1*(−) null genotype was observed in 12 COPD cases (6%) and 41 (20.5%) healthy individuals. The *GSTT1*(−) null genotype was significantly associated with mMRC in COPD patients (OR = 2.32, 95% CI = 1.05–5.14, *P* = 0.03). On the contrary the *GSTM1*(−) null genotype was present in 17 (8.5%) COPD cases and 73 (36.5%) healthy individuals. No significant relationship was found between *GSTM1*(−) and mMRC in COPD cases (OR = 1.97, 95% CI = 0.92–4.18, *P* = 0.07) (Table 4).

**TABLE 4:** Association of GST polymorphism with mMRC and CAT score for COPD risk.

Genotype	Controls (*n* (%)) (*n* = 200)	Cases (*n* (%)) (*n* = 200)	OR (95% CI)	*P*	AOR (95% CI)	*P*
mMRC < 2						
*GSTM1*						
*GSTM1*(+)	127 (63.5)	15 (7.5)	1.00 (reference)	–	–	–
*GSTM1*(−)	73 (36.5)	17 (8.5)	1.97 (0.92–4.18)	0.07	2.37 (0.96–5.84)	0.06
*GSTT1*						
*GSTT1*(+)	159 (79.5)	20 (10)	1.00 (reference)	–	1.00 (reference)	–
*GSTT1*(−)	41 (20.5)	12 (6)	2.32 (1.05–5.14)	**0.03**	1.61 (0.62–4.15)	0.32
mMRC ≥ 2						
*GSTM1*						
*GSTM1*(+)	127 (63.5)	112 (56)	1.00 (reference)	–	1.00 (reference)	–
*GSTM1*(−)	73 (36.5)	56 (28)	0.86 (0.56–1.33)	0.52	0.74 (0.36v1.50)	0.41
*GSTT1*						
*GSTT1*(+)	159 (79.5)	111 (55.5)	1.00 (reference)	–	1.00 (reference)	–
*GSTT1*(−)	41 (20.5)	57 (28.5)	1.99 (1.24–3.18)	**0.004**	4.13 (1.80–9.46)	**0.0008**
CAT < 10						
*GSTM1*						
*GSTM1*(+)	127 (63.5)	15 (7.5)	1.00 (reference)	–	1.00 (reference)	–
*GSTM1*(−)	73 (36.5)	17 (8.5)	1.97 (0.92–4.18)	0.07	2.37 (0.96–5.84)	0.06
*GSTT1*						
*GSTT1*(+)	159 (79.5)	20 (10)	1.00 (reference)	–	1.00 (reference)	–
*GSTT1*(−)	41 (20.5)	12 (6)	2.32 (1.05–5.14)	**0.03**	1.61 (0.62–4.15)	0.32
CAT ≥ 10						
*GSTM1*						
*GSTM1*(+)	127 (63.5)	112 (56)	1.00 (reference)	–	1.00 (reference)	–
*GSTM1*(−)	73 (36.5)	56 (28)	0.86 (0.56–1.33)	0.52	0.74 (0.36–1.50)	0.41
*GSTT1*						
*GSTT1*(+)	159 (79.5)	111 (55.5)	1.00 (reference)	–	1.00 (reference)	–
*GSTT1*(−)	41 (20.5)	57 (28.5)	1.99 (1.24–3.18)	**0.004**	4.13 (1.80–9.46)	**0.0008**

OR, crude odds ratio; AOR, adjusted odds ratio, evaluated by unconditional logistic regression and adjusted for age, sex and smoking. *P* < 0.05 was considered to be statistically significant.

In individuals having mMRC **≥** 2, the GSTT*1*(−) null genotype was reported in 57 (28.5%) COPD patients and 41 (20.5%) healthy individuals. A significant association was found between *GSTT1*(−) and mMRC in COPD cases (OR = 1.99, 95% CI = 1.24–3.18, *P* = 0.004). When adjusted with parameters like age, sex and smoking status, there was a strong correlation between *GSTT1*(−) null genotype and mMRC **≥** 2 for COPD risk (AOR = 4.13, 95% CI = 1.80–9.46, *P* = 0.0008). However, *GSTM1*(−) was present in 56 (28%) COPD cases and 73 (36.5%) healthy individuals and no correlation was found between *GSTM1*(−) and mMRC **≥** 2 for COPD risk (OR = 0.86, 95% CI = 0.56–1.33, *P* = 0.52) (Table 4).

The association of *GST* polymorphism and COPD Assessment Test (CAT) score was evaluated. Individuals were grouped into two categories suggested by the GOLD 2011 guidelines, that is, CAT score <10 and CAT score ≥10. In the first group of CAT score <10, the the *GSTT1*(−) null genotype was present in 12 (6%) COPD cases and 41 (20.5%) healthy individuals. A significant two‐fold increase in COPD risk was observed with the *GSTT1*(−) null genotype (OR = 2.32, CI = 1.05–5.14, *P* = 0.03). On the other hand, the *GSTM1*(−) genotype was observed in 17 (8.5%) COPD cases and 73 (36.5%) healthy individuals. No significant relationship was found between *GSTM1*(−) and CAT score <10 in COPD cases (OR = 1.97, 95% CI = 0.92–4.18, *P* = 0.07) (Table 4).

In the second group, individuals having a CAT score **≥**10, the *GSTT1*(−) null genotype was reported in 57 (28.5%) COPD patients and 41 (20.5%) healthy individuals. A significant association was observed between the *GSTT1(−)* null genotype and CAT score among COPD patients (OR = 1.99, 95% CI = 1.24–3.18, *P* = 0.004). When adjusted for parameters like age, sex and smoking status, a strong correlation between *GSTT1*(−) null genotype and CAT score ≥10 for COPD risk was observed (AOR = 4.13, 95% CI = 1.80–9.46, *P* = 0.0008). However, *GSTM1*(−) was present in 56 (28%) COPD cases and 73 (36.5%) healthy individuals and no correlation was found between *GSTM1*(−) and CAT score ≥10 for COPD risk (OR = 0.86, 95% CI = 0.56–1.33, *P* = 0.52) (Table 4).

### 3.6 Association of *GST* polymorphism with GOLD severity and GOLD ‘ABCD’ symptom‐based assessment for COPD risk

We also assessed the relationship between *GST* polymorphism and GOLD severity. Subjects were divided into two groups: group A included mild (FEV1 ≥ 80% predicted) and moderate (50% ≤ FEV1 < 80% predicted) COPD cases of airflow obstruction, and group B included severe (30% ≤ FEV1 < 50% predicted) and very severe (FEV1 < 30% predicted) COPD cases of airflow limitation.

In group A, the *GSTT1*(−) null genotype was found in 26 (13%) COPD cases and 41 (20.5%) healthy individuals. The *GSTT1*(−) genotype was found to have a significant relationship with GOLD severity for susceptibility to COPD (OR = 1.93, 95% CI = 1.08–3.47, *P* = 0.02). However, there was no association between *GSTM1*(−) and GOLD severity of airway limitation in COPD cases (Table 5).

**TABLE 5:** Association of GST polymorphism with GOLD severity for COPD risk and GOLD ‘ABCD’ symptom‐based assessment.

Genotype	Controls (*n* (%)) (*n* = 200)	Cases (*n* (%)) (*n* = 200)	OR (95% CI)	*P*	AOR (95% CI)	*P*
GOLD severity (group A—mild + moderate)						
*GSTM1*						
*GSTM1*(+)	127 (63.5)	45 (22.5)	1.00 (reference)	–	1.00 (reference)	–
*GSTM1*(−)	73 (36.5)	33 (16.5)	1.27 (0.74–2.17)	0.37	1.34 (0.63–2.86)	0.44
*GSTT1*						
*GSTT1*(+)	159 (79.5)	52 (26)	1.00 (reference)	–	1.00 (reference)	–
*GSTT1*(−)	41 (20.5)	26 (13)	1.93 (1.08–3.47)	**0.02**	1.93 (0.84–4.47)	0.12
GOLD severity (group B—severe + very severe)						
*GSTM1*						
*GSTM1*(+)	127 (63.5)	82 (41)	1.00 (reference)	–	1.00 (reference)	–
*GSTM1*(−)	73 (36.5)	40 (20)	0.84 (0.52–1.36)	0.49	0.85 (0.40–1.80)	0.67
*GSTT1*						
*GSTT1*(+)	159 (79.5)	79 (39.5)	1.00 (reference)	–	1.00 (reference)	–
*GSTT1*(−)	41 (20.5)	43 (21.5)	2.11 (1.27–3.50)	**0.003**	3.33 (1.42–7.81)	**0.005**
GOLD A						
*GSTM1*						
*GSTM1*(+)	127 (63.5)	14 (7)	1.00 (reference)	–	1.00 (reference)	–
*GSTM1*(−)	73 (36.5)	15 (7.5)	1.86 (0.85–4.07)	0.11	2.26 (0.90–5.65)	0.08
*GSTT1*						
*GSTT1*(+)	159 (79.5)	18 (9)	1.00 (reference)	–	1.00 (reference)	–
*GSTT1*(−)	41 (20.5)	11 (5.5)	2.36 (1.03–5.40)	**0.04**	1.63 (0.63–4.32)	0.30
GOLD B						
*GSTM1*						
*GSTM1*(+)	127 (63.5)	98 (49)	1.00 (reference)	–	1.00 (reference)	–
*GSTM1*(−)	73 (36.5)	48 (24)	0.85 (0.54–1.33)	0.48	0.80 (0.39–1.66)	0.56
*GSTT1*						
GSTT1(+)	159 (79.5)	93 (46.5)	1.00 (reference)	–	1.00 (reference)	–
*GSTT1*(−)	41 (20.5)	53 (26.5)	2.2 (1.36–3.57)	**0.001**	5.06 (2.11–12.11)	**0.0003**
GOLD C						
*GSTM1*						
*GSTM1*(+)	127 (63.5)	1 (0.5)	1.00 (reference)	–	1.00 (reference)	–
*GSTM1*(−)	73 (36.5)	2 (1)	3.4 (0.31–39.03)	0.31	4.37 (0.33–57.39)	0.26
*GSTT1*						
*GSTT1*(+)	159 (79.5)	2 (1)	1.00 (reference)	–	1.00 (reference)	–
*GSTT1*(−)	41 (20.5)	1 (0.5)	1.93 (0.17–21.91)	0.59	1.39 (0.10–18.53)	0.80
GOLD D						
*GSTM1*						
*GSTM1*(+)	127 (63.5)	14 (7)	1.00 (reference)	–	1.00 (reference)	–
*GSTM1*(−)	73 (36.5)	8 (4)	0.99 (0.39–2.48)	0.98	0.89 (0.25–3.10)	0.85
*GSTT1*						
*GSTT1*(+)	159 (79.5)	18 (9)	1.00 (reference)	–	1.00 (reference)	–
*GSTT1*(−)	41 (20.5)	4 (2)	0.86 (0.27–2.68)	0.79	0.56 (0.13–2.44)	0.44

OR, crude odds ratio; AOR, adjusted odds ratio, evaluated by unconditional logistic regression and adjusted for age, sex and smoking. *P* < 0.05 was considered to be statistically significant.

In group B, the *GSTT1*(−) null genotype was found in 43 (21.5%) COPD cases and 41 (20.5%) healthy individuals with a two‐fold risk of severity in COPD patients, and the association was found to be significant (OR = 2.11, 95% CI = 1.27–3.50, *P* = 0.003). After adjusting for various covariates like age, sex and smoking status, there was an associated risk between *GSTT1*(−) null individuals and GOLD severity in COPD patients (AOR = 3.33, 95% CI = 1.42–7.81, *P* = 0.005) However, no association was found between *GSTM1*(−) and GOLD severity of airway limitation in COPD cases (Table 5).

GOLD ABCD is a refined evaluation tool that offers symptom burden and exacerbation risk information that can be used to guide treatment. According to GOLD 2011 guidelines, we divided our COPD cases into four groups designated A, B, C and D. We evaluated the relationship between GST polymorphism and GOLD ‘ABCD’ symptom‐based assessment.

In category A, 11 (5.5%) COPD cases and 41 (20.5%) healthy individuals had the *GSTT1*(−) null genotype. A significant correlation was observed between *GSTT1*(−) and GOLD A category among COPD cases (OR = 2.36, 95% CI = 1.03–5.40, *P* = 0.04). In category B, the *GSTT1*(−) null genotype was present in 53 (26.5 %) COPD cases and 41 (20.5%) healthy individuals. A significant association was found between *GSTT1*(−) and GOLD B category with two‐fold risk in patients harbouring the *GSTT1*(−) null genotype (OR = 2.2, CI = 1.36–3.57, *P* = 0.001). A strong correlation of *GSTT1*(−) with the GOLD B category was observed after adjusting for parameters like age, sex and smoking status with a five‐fold increase in COPD risk (AOR = 5.06, 95% CI = 2.11–12.11, *P* = 0.0003). On the other hand, *GSTT1*(−) showed no correlation with group C and D among COPD cases. Furthermore, the *GSTM1*(−) null genotype and any of the groups mentioned above showed no significant association (Table 5).

### 3.7 Correlation of *GST* polymorphism and COPD duration

We studied the association between *GST* polymorphism and COPD duration. COPD duration was classified into four categories, that is, <2 years, 2–5 years, 5–10 years and >10 years. In the <2 years category, the *GSTT1*(−) genotype was reported in 28 (14%) COPD cases and 41 (20.5%) healthy participants. There was a significant association of *GSTT1*(−) and COPD duration (OR = 2.17, 95% CI = 1.22–3.86, *P* = 0.008). In the 2–5 years category, 17 (8.5%) COPD patients and 41 (20.5%) healthy individuals had the *GSTT1*(−) genotypes. After adjusting with covariates like age, sex and smoking status, a significant relationship was observed between *GSTT1*(−) and COPD 2–5 years (AOR = 2.76, 95% CI = 1.03–7.33, *P* = 0.04). In the >10 years category, the *GSTT1*(−) genotype was present in 16 (8%) COPD cases and 41 (20.5%) healthy individuals. A significant correlation was observed between *GSTT1*(−) and COPD duration (OR = 2.82, 95% CI = 1.39–5.85, *P* = 0.005). However, *GSTT1*(−) showed no correlation with the 5–10 years category among COPD cases. Moreover, *GSTM1*(−) null genotype and any of the groups as mentioned above showed no significant association (data not shown).

## 4 DISCUSSION

Our lungs are subjected to atmospheric pollution, irritants, noxious particles, allergens and pathogens daily, which can trigger inflammatory responses and the production of endogenous oxidants. This may further result in chronic inflammation, tissue injury and remodelling. Inhaled xenobiotic compounds are rapidly absorbed and metabolized by the phase I and II enzymes critical in detoxification, including the glutathione‐conjugating enzymes GSTs (Van de Wetering et al., 2021). When *GST* genes are absent or there are *GST* gene variants, there is a loss of related enzyme activity and accumulation of foreign substances, which can cause many diseases, including COPD.

The case–control research approach was used in this experimental investigation; our COPD patients had a mean age of 59.3 ± 9.9 years, while healthy individuals had a mean age of 47 ± 15.6 years. Similar results were obtained by Dimov et al. (2008), with a mean age of 67 years for COPD cases and 57 years for healthy individuals (Dimov et al., 2008). Gaspar et al. (2004) reported a mean age of 64.3 years for COPD cases and 31.9 years for healthy individuals (Gaspar et al., 2004).

The mean BMI was 21.7 kg/m^2^ for COPD cases and 25.3 kg/m^2^ for healthy individuals. A study by Arja et al. (2013) revealed a mean BMI of 19.7 in COPD cases and a mean BMI of 21.5 kg/m^2^ in healthy individuals. Another study by Begum et al. (2014) showed a mean BMI of 19.7 kg/m^2^ among COPD cases and a mean BMI of 28.4 kg/m^2^ among healthy individuals (Begum et al., 2014). COPD patients’ BMI is comparatively lower than healthy individuals due to increased metabolic rate and decreased absorption.

The mean pack‐years of smoking in our study was 49.6. Lakhdar et al. (2010) and Begum et al. (2014) found mean pack‐years of 51.8 ± 29.4 and 88.2 ± 19.8, respectively. However, a study done by Faramawy et al. (2009) and Mehrotra et al. (2010) found that the average pack‐years in COPD cases was 35.3 ± 7.0 and 20.8 ± 4.7, respectively (Begum et al., 2014; Faramawy et al., 2009; Lakhdar et al., 2011; Mehrotra et al., 2010). This study supports our results, showing that the development of COPD can occur even with lower pack‐years of smoking.

Most of our COPD patients belonged to GOLD stage 2 (35.5%) and 3 (39.5%). Begum et al. (2014) found similar results, reporting moderate and severe COPD in 121 of patients. Our finding indicates that patients reporting to tertiary hospitals in India are in the late stages of COPD with severe symptoms. Reporting the contrary, results were found in a study by Ekberg‐Aronsson et al. (2005) and Begum et al. (2014). This could be because patients in developed countries have better education, awareness and health infrastructure, leading to earlier disease detection and immediate treatment.

The frequency of null genotypes for *GSTT1* and *GSTM1* varies among ethnic groups. The variation in gene polymorphisms may be related to different metabolizing enzyme activities and dominant functional enzymes against oxidative stress in different races (Cheng et al., 2004). Ethnic differences in COPD prevalence are difficult to distinguish from environmental parameters (Yim et al., 2000). A study suggested that 60% of Asians, 40% of Africans and 20% of Caucasians show *GSTT1* enzyme deficiency (Zhang et al., 2014). Similar results were obtained from our study, which showed the prevalence of the *GSTT1*(−) genotype to be 34.5% among COPD cases compared to 20.5% among healthy individuals. Nevzorova et al. (2012), Kukkonen et al. (2011), Mehrotra et al. (2010) and Thakur et al. (2011) also reported similar results. Our research arch emphasizes the fact that the *GSTT1*(−) null genotype is a critical factor in developing COPD; however, a few studies have yielded contradictory results (Dimov et al., 2008; Gaspar et al., 2004; Hemimi, 2008; Yim et al., 2000; Žuntar et al., 2014).

No evident association between the *GSTM1*(−) null allele and COPD susceptibility was found. Our findings contradict previous research (Ahmad et al., 2016; Dey et al., 2014; Faramawy et al., 2009; Gaspar et al., 2004; Kant Shukla et al., 2013).

The combined effect of the *GSTM1*(−) null and *GSTT1*(−) null genotype was a two‐fold COPD risk, but the association was insignificant, which was similar to the investigation conducted by Mehrotra et al. (2010). The combinatorial association of *GSTM1*(+) and *GSTT1*(−) null genotypes increased the susceptibility to COPD by two‐fold.

Per our understanding, our study is the first to report the correlation of *GST* polymorphism with mMRC grading and CAT score. The null genotype of *GSTT1* showed a strong association with higher mMRC and CAT scores. It thus shows that individuals lacking the *GSTT1* gene are more likely to experience escalating breathlessness and higher CAT scores.

The *GSTT1*(−) null allele was found to be associated with both males and females for COPD susceptibility. Females with the *GSTT1*(−) null genotype were at a higher risk of developing COPD than males with the same gene deletion. Women smokers were roughly 50% more likely than men to develop COPD in a systematic review despite smoking fewer cigarettes (Gan et al., 2006).

A strong correlation between *GST* polymorphism and GOLD severity was found for the *GSTT1*(−) null genotype. Research done by Ahmad et al. (2016) proposed that the prevalence of the *GSTM1*(−) null genotype was greater in patients with severe COPD (Ahmad et al., 2016).

A strong association of the *GSTT1*(−) null allele with the GOLD B category for susceptibility to COPD was found. This suggests that the patients having this allele have higher symptom severity but lower exacerbation risk. No other study has made this correlation before, and more data are needed to get in‐depth knowledge of this association. A significant correlation between *GSTT1*(−) and COPD duration was found, indicating that patients with a longer duration of COPD have lost the activity of the *GSTT1* gene.

With the rapidly increasing number of COPD cases and high mortality rates worldwide, greater attention should be paid to treating and preventing the disorder. Currently, no medication has shown the best results against the pulmonary damage caused by COPD. Genetic markers can play a massive role in detecting diseases early and preventing them from progressing to severe stages. Our research has made one such attempt to find a suitable genetic marker for COPD in North Indian patients. According to our findings, the *GSTT1*(−) null genotype can be used as a genetic biomarker. *GSTT1* deletion was also linked to increased risk for developing COPD and linked to higher GOLD grading. A more extensive population study can clarify the relationship between the homozygous null *GSTT1* gene and other xenobiotic enzymes as risk factors for COPD development and pathogenesis.

## AUTHOR CONTRIBUTIONS

Conceptualization: Sidhartha Singh, Vishal Chopra, Kranti Garg; Harsh Yadav, Karan Sharma, Nidhi Girdhar collected patient sample and information. Depanshi Pandit, Parul Sharma, and Sidhartha Singh performed molecular and statistical analyses and wrote the first paper draft; Vishal Chopra, Kranti Garg, Harsh Yadav, Karan Sharma, Nidhi Girdhar provided all clinical data of patients. Vishal Chopra, Kranti Garg, Sidhartha Singh reviewed the final version of paper. All authors have read and approved the final version of this manuscript and agree to be accountable for all aspects of the work in ensuring that questions related to the accuracy or integrity of any part of the work are appropriately investigated and resolved. All persons designated as authors qualify for authorship, and all those who qualify for authorship are listed.

## CONFLICT OF INTEREST

All of the authors of this manuscript report that they have no conflict of interest.

## FUNDING INFORMATION

None.

## Data Availability

All data shall be made available if needed.
